# Positional Obstructive Sleep Apnea Syndrome in Elderly Patients

**DOI:** 10.3390/ijerph17031120

**Published:** 2020-02-10

**Authors:** Giannicola Iannella, Giuseppe Magliulo, Cristina Anna Maria Lo Iacono, Giulia Bianchi, Antonella Polimeni, Antonio Greco, Andrea De Vito, Giuseppe Meccariello, Giovanni Cammaroto, Riccardo Gobbi, Marco Brunori, Milena Di Luca, Filippo Montevecchi, Annalisa Pace, Irene Claudia Visconti, Claudia Milella, Carmen Solito, Stefano Pelucchi, Luca Cerritelli, Claudio Vicini

**Affiliations:** 1 Department of ‘Organi di Senso’, University “Sapienza”,Viale dell’Università, 33, 00185 Rome, Italy; giuseppe.magliulo@uniroma1.it (G.M.); antonio.greco@uniroma1.it (A.G.); annalisapace90@gmail.com (A.P.); ireneclaudia.visconti@gmail.com (I.C.V.); claudiamilella@hotmail.it (C.M.); carmensolito7@gmail.com (C.S.); 2 Department of Head-Neck Surgery, Otolaryngology, Head-Neck and Oral Surgery Unit, Morgagni Pierantoni Hospital, Via Carlo Forlanini, 34, 47121 Forlì, Italy; dr.andrea.devito@gmail.com (A.D.V.); drmeccariello@gmail.com (G.M.); giovanni.cammaroto@hotmail.com (G.C.); dr.riccardogobbi@gmail.com (R.G.); claudio@claudiovicini.com (C.V.); 3 Department of Cardiovascular, Respiratory, Nephrologic, Anaesthesiologic and Geriatric Sciences, Sapienza University, Viale dell’Università, 33, 00185 Rome, Italy; cristina.loiacono@uniroma1.it (C.A.M.L.I.); marco.brunori@uniroma1.it (M.B.); 4 Department ENT & Audiology, University of Ferrara, Via Savonarola, 9, 44121 Ferrara, Italy; giuwhites91@gmail.com (G.B.); stefano.pelucchi@unife.it (S.P.); luca.cerritelli.bo@gmail.com (L.C.); 5 Department of Oral and Maxillo Facial Sciences, University “Sapienza”, Viale dell’Università, 33, 00185 Rome, Italy; antonella.polimeni@uniroma1.it; 6 Department of ENT, University of Catania, Via S. Sofia, 78, 95125 Catania, Italy; milenadiluca@hotmail.it; 7 ENT Unit Villa Serena, Via del Camaldolino, 8, 47121 Forlì, Italy; filippomontevecchi72@gmail.com

**Keywords:** obstructive sleep apnea, positional sleep apnea, aging effects, polysomnography

## Abstract

*Background* The purpose of this study was to evaluate the prevalence of position-dependent obstructive sleep apnea (POSA) in elderly patients (≥65 years old). Adult (range 19-65 years old) and elderly patients were also compared in order to show differences in the incidence of POSA between these two groups of patients. *Methods* A prospective bi-center study was performed between January 2018 and May 2019. A total of 434 participants underwent polysomnography (PSG) study at home (Embletta MPR). Body position during the PSG recordings was determined. Patients were subdivided in two groups: those aged between 19 and 65 years old (adult patients) and ≥65 years old (elderly patients). POSA patients were defined using Cartwright’s system, Bignold classification, and the new Amsterdam Positional OSA Classification (APOC). *Results* The prevalence of POSA in elderly patients differed according to the classification system used: 49.3% using Cartwright’s classification system, 20.5% with the Bignold classification, and 22.6%, 38.9%, and 5.4% of APOC 1, APOC 2, and APOC3 sub-classes were respectively identified for the APOC classification system. No difference between adult and elderly patients regarding the prevalence of POSA was observed. No statistical differences emerged between the two groups of patients in terms of supine (*p* = 0.9) and non-supine AHI (*p* = 0.4). *Conclusions* A significant number of elderly patients could be considered treatable with positional therapy according to the APOC classification. However, the efficacy and applicability of positional therapy in elderly patients must be confirmed by further research.

## 1. Introduction

Obstructive sleep apnea (OSA) syndrome is one of the most common sleep-disorder breathing (SDB) conditions [1,2]. It is characterized by the reduction (hypopnea) or complete cessation (apnea) of airflow in the upper airways during the night [3,4].

In accordance with the American Academy of Sleep Medicine (AASM) [5,6], the diagnosis and classification of OSA was made on the basis of the average number of apneas and hypopneas in relation to the hours of sleep. This ratio is expressed by the apnea–hypopnea index (AHI). Patients with an AHI <5 are considered normal subjects or simple snorers if night snoring is present. Patients with an AHI ≥5 to <15 are classified as mild OSA, those with an AHI ≥15 to <30 are considered moderate OSA, and patients with an AHI ≥30 are classified as severe OSA.

The incidence of OSA is often underestimated, affecting between 2% and 5% of the middle-aged population, but this percentage could change with aging [7,8,9]. Different studies have estimated OSA incidence rates from 5.6% to 60% in people over 65 [10,11,12] and aging has been positively related with an increase in OSA incidence [13,14,15,16,17]. In a study comprising 5615 men and women between 40–98 years of age, sleep apnea was found to be most frequent in subjects aged 60 years or older (approximately 50% had an AHI of 5–14, and approximately 20% had an AHI ≥15) [18].

In patients with OSA, the frequency and duration of apneas could be influenced by body position. Patients, with variations in the number/duration of apneas/hypopneas related to changes in their sleeping position, have been defined as position-dependent OSA (POSA) patients [19,20,21]. 

To date, various classification systems for POSA have been proposed in the literature. Cartwright’s classification was the first of these to be proposed in the literature and is still the form most widely used in the world in clinical practice. Cartwright’s definition of POSA patients required a difference of 50% or more in AHI between supine and non-supine positions. The limitation of Cartwright’s system is that it does not take into consideration the amount of sleeping time spent in the supine and non-supine positions. Considering this classification system alone, patients who sleep only a few minutes in a non-supine position may be wrongly classified as POSA patients. To overcome this limitation, various modified versions of Cartwright’s criteria and alternative POSA classification systems have been proposed over the last years [22,23]. Bignold et al. were the first to propose an alternative classification taking into account the amount of sleeping time in a supine and non-supine positions. According to this classification system, patients with ≥20 min of sleep in supine and non-supine postures could be considered POSA according to the reduction of AHI >50%.

Finally, a new classification system called the Amsterdam Positional OSA Classification (APOC) has recently been proposed [24]. It considers the total sleep time (TST) in both the best sleeping position (BSP) and worst sleeping position (WSP), as well as the grade of AHI reduction in the best sleeping position. Authors claimed that this system is helpful not only for identifying patients with POSA but is also effective in determining suitable candidates for position therapy. The authors also proposed the use of this classification system to facilitate the collection of data across multiple centers and for the comparison of results across studies.

Over the last years, an increasing amount of papers have been published regarding obstructive sleep apnea in elderly patients. Several authors have reported that aging could be associated with changes in OSA-related parameters (AHI, mean peripheral oxygen saturation [SpO_2_], reduction of daytime sleepiness, etc.) and duration and type of sleep [25,26,27,28]. Moreover, as recently reported by Vicini et al. and Zhao et al., the number of obstruction sites and the collapse pattern may change over time due to changes in pharyngeal anatomy, redistribution of body fat, and/or an increase in the laxity of the oro/hypo-pharyngeal muscular structures, which are known to occur with aging [29,30]. 

These changes in the structure and function of the upper airway, pharyngeal collapsibility, and type and pattern of collapse could have an effect on the collapse of the pharyngeal wall in the lateral position. Is it therefore possible that the prevalence of positional OSA is different in elderly patients?

In a recent study, Vicini et al. compared drug-induced sleep endoscopy (DISE) findings and did not observe any differences between elderly and young patients in the supine and lateral positions. However, the study comprised a limited group of patients and polysomnographic data regarding POSA were not analyzed [29].

This study was designed to evaluate the incidence of POSA in elderly patients and to identify any differences between adult patients (ranged 19–65 years old) and elderly patients (≥65 years) in terms of the prevalence of position-dependent OSA. 

Three classification systems (Cartwright, Bignold, and APOC) were used in this study to report the results of the prevalence of POSA in the elderly population and to compare the results between these patients and the group of adult patients. The Cartwright classification, despite the limitations described above, was chosen because it is still the form most commonly used in the world today. The Bignold classification was considered to avoid over- or underestimations of the incidence POSA possible using Cartwright’s classification alone. Finally, the APOC classification was chosen because it is currently considered the most valid method for identifying POSA patients and its subtypes. Unfortunately, its use is not yet so widespread.

## 2. Materials and Methods 

### 2.1. Enrollment of Patients

This prospective bi-center study was performed at the Otolaryngology, Head and Neck and Oral Surgery Department of the Morgagni Pierantoni Hospital in Forlì, Italy, and at the ‘Organi di Senso’ Department of ‘Sapienza’ University in Rome, Italy, between January 2018 and May 2019.

Subjects eligible for the study were initially selected from patients referred to these departments with a suspicion or a diagnosis of OSA. All these patients underwent polysomnography (PSG) after the initial otolaryngologic examination.

In accordance with the American Academy of Sleep Medicine (AASM) [31], the diagnosis and classification of OSA was performed on the basis of the apnea + hypopnea index (AHI). 

In accordance with the existing literature, in this study an age over 65 years was taken to be indicative of elderly patients [29,32]. Patients were subdivided in two groups: those aged between 19 and 65 years old (adult patients) and ≥65 years old (elderly patients).

Sex and body mass index (BMI) were initially evaluated for each patient of the study.

This research study was performed in accordance with the principles of the Declaration of Helsinki and approved by the local ethics committee. Informed consent was obtained from all individual participants included in the study.

### 2.2. Inclusion and Exclusion Criteria

Patients younger than 18 years, as well as pediatric patients, were excluded from the study. Simple snorers, according to PSG results (AHI < 5/h), were excluded from the study. Patients submitted to surgical treatment for OSA or who had been submitted to other head–neck surgical procedures were also excluded from the study in order to evaluate POSA prevalence without bias of the previous surgery. Finally, patients with cognitive impairment or neurological diseases and patients receiving pharmacological treatment for OSA or drugs with an impact on the cognitive function were not considered eligible for the study.

### 2.3. Polysomnographic Data

All participants of the study underwent a home sleep apnea test, polysomnography type III (HSAT; Embletta MPR) as defined in the AASM rules [24,31]. The following parameters were recorded during the sleep study: respiratory movement and airflow, heart rate, arterial oxygen saturation, patient’s position, and sleep time. Body position during the PSG recordings was determined using a titanium built-in three dimensional accelerometer (XYZ) with a sampling rate of 32 Hz. This sensor provided the following position outputs: supine, right, left, prone, or upright. 

The PSG type III device used for the diagnosis of OSA in this study did not record the signals needed to determine sleep stages or sleep disruption (electroencephalogram).

We asked individuals who were currently receiving treatment for sleep-disordered breathing (Continuous positive airway pressure [CPAP], oral appliance, nose device) to discontinue their treatment 1 week before the sleep recording.

Two trained technicians scored polysomnographic recordings manually using Remologic software (version 5.1.1, Embla). 

Respiratory events were scored using the latest 2012 AASM criteria—apnea was defined as a drop of at least 90% of airflow from baseline, lasting 10 seconds or longer. Hypopnea was defined as a ≥30% drop of airflow lasting at least 10 seconds, associated with either an arousal or a ≥3% O_2_ saturation drop [24,31]. 

The average number of apneas and hypopneas per hour of sleep (AHI) was calculated. 

Two co-authors of the study (G.I., G.B.) reviewed every recording, and a second sleep expert did random quality checks. 

The apnea–hypopnea index (AHI), supine-AHI, non supine-AHI, oxygen desaturation index (ODI), supine-ODI, non supine-ODI, mean SpO_2_, and mean time in supine and non-supine positions were collected for each patient’s PSG study. The PSG data of both subgroups of patients were analyzed and compared.

In accordance with the American Academy of Sleep Medicine (AASM) [31], classification of OSA was made on the basis of the apnea–hypopnea index (AHI). Patients were classified into mild OSA (AHI ≥ 5 and <15), moderate OSA (AHI ≥ 15 and <30), and severe OSA (AHI ≥ 30). The simple snorers according to PSG results (AHI was <5/h) were excluded from the study.

### 2.4. POSA Diagnosis and Classifications 

POSA patients were defined using Cartwright’s system, Bignold classification, and the new Amsterdam Positional OSA Classification (APOC). Cartwright’s definition of POSA patients requires a difference of 50% or more in AHI between supine and non-supine positions [33]. The Bignold classification provides the following criteria for defining a position-dependent patient: overall AHI ≥15/h, supine AHI ≥ twice the non-supine AHI, ≥20 min of sleep in supine and non-supine postures [34]. Patients were considered to be position-dependent according to the APOC if the following criteria were fulfilled: diagnosis of OSA according to the American Academy of Sleep Medicine (AASM) criteria; greater than 10% of the total sleep time (TST) in both the best sleeping position (BSP) and worst sleeping position (WSP). Patients are diagnosed APOC 1 if AHI < 5 was present in BSP; patients are diagnosed APOC II if the AHI in the BSP falls into a lower OSA severity category than the overall AHI; patients with an overall AHI ≥ 40 AHI in BSP > 25% reduction compared to overall AHI were defined as APOC III [22,23]. 

### 2.5. Statistical Analysis

The χ^2^ test was employed to evaluate the differences in the prevalence of POSA between the two groups of patients using the different classification criteria. The Student’s *t*-test was used for comparing the clinical and polysomnographic factors (age, body mass index, AHI, AHI in supine position, AHI in non-supine position, mean time in supine position, mean time non-supine position, total time of sleep, mean SpO_2_, oxygen desaturation index). A *p*-value of <0.05 was taken as the threshold of statistical significance.

A multi-variate analysis of the different risk factors correlated with POSA was performed. An odds ratio (OR) <1 was considered indicative of a negative correlation between the risk factor examined and the POSA.

## 3. Results

### 3.1. Clinical Results

A total of 146 patients with a diagnosis of OSA aged ≥65 years old were enrolled in the study. Morever, 288 patients aged between 19 and 65 years old were enrolled in a control group of adult patients. Patient characteristics (age, BMI, and sex) and PSG data (AHI, mean SpO_2_, oxygen desaturation index, total time of sleep, etc.) of both groups of patients have been summarized in Table 1.

The mean age of patients over 65 years was 72.4 years (range of 65–90), whereas the mean age of the control group was 51.4 (range of 19–64) years-old (*p* = 0.0001). No differences in sex distribution emerged between the two groups of patients.

The mean BMI of elderly patients (mean value of 29.9) was found to be lower than that of adult patients (mean value of 31.1), although no statistical difference emerged (*p* = 0.06) between the two groups of patients. 

Regarding the severity of OSA, a mean AHI of 28.3 and 27.4 emerged in elderly and adult patients, respectively. Comparing these two mean values, no statistical difference emerged (*p* = 0.6). Similarly, no differences were observed in a comparison of the distribution of elderly and adult patients within the different AHI sub-classes (*p* > 0.05 for each class of OSA severity) (Table 2). Similarly, the mean ODI and mean SpO_2_ did not show any statistical difference between the two patient groups (Table 1). No statistical differences emerged between the two groups of patients in terms of total time of sleep (*p* = 0.4).

### 3.2. POSA Results

In elderly patients the mean values of supine AHI and non-supine AHI were found to be 37.7 and 21.7, respectively. Similar values emerged in the adult patients with a supine AHI value of 38 and a non-supine AHI of 19.9. No statistical differences emerged between the two groups of patients in terms of supine (*p* = 0.9) and non-supine AHI (*p* = 0.4). 

The prevalence of POSA in elderly patients differed according to the classification system used (Table 3).

Using Cartwright’s classification system, 49.3% of patients of the elderly group were defined as POSA, whereas in the group of adult patients, 51.3% of patients were classified positive using the same classification system. No differences in POSA prevalence emerged between the two groups of patients when Cartwright’s classification (*p* = 0.7) was used.

Estimating positionality with the Bignold classification, the prevalence of POSA was 20.5% in the elderly group. A similar value emerged in the adult group of patients with a prevalence of 22.9%. No statistical differences in the prevalence of POSA were observed using this type of classification system either (*p* = 0.6).

Finally, in elderly patients with the use of the APOC classification system, 22.6%, 38.9%, and 5.4% of APOC 1, APOC 2, and APOC3 sub-classes were identified, respectively. No difference emerged between elderly and adult patients in the APOC sub-class distribution (*p* > 0.05 for each APOC class).

**Figure 1:**
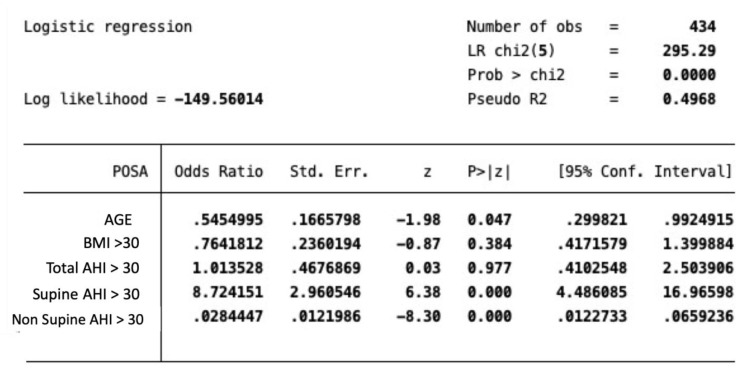
Multi-variable analysis of possible risk factors for POSA.

Finally, a multi-variate analysis of the different risk factors associable with POSA are reported in Figure 1.

For age, an odds ratio of 0.5 was indicative of a negative correlation between aging and POSA. A supine AHI > 30 has been proven as a risk factor for POSA (odds ratio 8.7).

## 4. Discussion

The usual definition of positional sleep apnea is a supine apnea–hypopnea index (AHI) that is greater than twice the non-supine AHI, as initially described by Cartwright in 1984. Using this original definition, literature studies have shown that in middle-aged patients, the incidence of POSA varies from 56% to 71.4% with a mean of 62.6% ± 6.8% (SD) [35,36,37]. However, the major limitation of Cartwright’s system is that it is a failing classification modality that does not take into consideration the time of sleep in the supine and non-supine positions. There have been studies that have measured the POSA incidence data with a much stricter definition (ratio of the supine AHI and non-supine AHI greater than 2, along with a non-supine AHI less than 10 events per hour) [33]. With this strict definition, the incidence of positional sleep apnea was substantially lower than the original definition and ranged from 23% to 27%. In the study by Mador et al., the prevalence of POSA in middle-aged patients using the lenient Cartwright definition was 58% and decreased to 27%, applying a more stricter classification system [38]. Recently, Frank et al [22] introduced a new classification system (Amsterdam Positional OSA Classification), which ideally should identify suitable candidates for position therapy. The authors also proposed the use of this classification system to facilitate collection of data across multiple centers and comparison of results across studies. APOC I are the patients who theoretically can be cured with the use of the only positional therapy (PT). APOC II are patients who theoretically can decrease their OSA severity category via treatment with PT, rendering other treatment options available. Finally, those patients who are classified as APOC III with an overall AHI ≥40 are those who can theoretically achieve a >25% reduction of their AHI with PT only, thereby improving compliance of existing therapies.

It is estimated that OSA affects up to 9% of men and 4% of women, but it has been observed that the prevalence may be even higher in elderly people [39]. One of the first literature studies analyzing the development of SBD in the elderly was conducted by Ancoli et al. [15]. They analyzed 427 elderly people over 65 years of age who were suffering from OSA, and showed that 24% of them presented an apnea/hypopnea index (AHI) greater than 5 and that 62% had a respiratory disturbance, with a respiratory disorder index score (RDI) ≥10. Moreover, as suggested by different authors, it seems that there is an aging effect on the OSA severity. Regarding this aspect, Vicini et al., using a regression analysis, observed an increase in AHI as patients’ age increased (*p* = 0.03) [29].

Along with this established evidence, there is a common idea that the positional effect in OSA patients seems to decrease as age increases, and thus adult patients have a higher probability of having positional sleep apnea. However, little evidence on this topic is reported in the literature, and no papers analyzing the prevalence of POSA in elderly patients and/or the difference between adult and elderly patients in terms of the prevalence of POSA have been published. Given the high prevalence of OSA syndrome in the elderly population and considering the increase in the average age of the world population, it is important to understand whether there are changes in the prevalence of POSA in people over 65 years of age.

We estimated the incidence of POSA in elderly patients (≥65 years old) by means of three different classification systems in order to obtain data that are a true expression of the prevalence of POSA in this class of patients, and to avoid over- or underestimations of the POSA prevalence that could be the result of using only the classification of Cartwright. Moreover, the APOC classification was chosen because it is currently considered the most valid method for identifying POSA patients and its subtypes.

In our study, the prevalence of POSA in elderly patients differed depending on the classification system used. Using Cartwright’s classification system, 49.3% of elderly patients were defined as POSA. When a more stringent system of classification was used (like that of Bignold, which takes into account the time spent in the supine and non-supine positions) a reduction of POSA to 27% in the elderly was observed. Finally, in elderly patients, with the use of the APOC classification system, 29.8%, 20.4%, and 6.9% of APOC 1, APOC 2, and APOC 3 sub-classes were identified, respectively.

In this study, we also estimated the prevalence of POSA in a group of adult OSA patients in order to compare results regarding POSA prevalence between adult and elderly patients.

In the group of adult patients, 51.3% were classified positive with the Cartwright’s classification system. These data were similar to that of 58% of POSA in middle-aged patients reported by Mador et al. [38]. Estimating positionality using the Bignold classification, a prevalence of 22.9% of POSA emerged in the adult group. A similar value emerged in the study of Mador et al., with an prevalence of 27% in POSA patients using a more stringent system of classification such as the Bignold form. Finally, in adult patients with the use of the APOC classification system, 29.8%, 20.4%, and 6.9% of APOC 1, APOC 2, and APOC3 sub-classes were identified, respectively.

In our study, no differences emerged regarding POSA prevalence in elderly and adult patients. All three different classification systems showed similar prevalence of POSA in adult and elderly patients (*p* > 0.05 for each classification system). Moreover, confirming the results of this study, it should be noted that no statistical difference emerged between the two groups of patients in terms of mean supine (*p* = 0.9) and non-supine AHI (*p* = 0.4). 

One of the advantages of this study was the high number of patients analyzed. Moreover, using different classification systems, we limited possible biases/deficiency of each single classification system. Similar results obtained using the different system of classification confirmed the data observed in the study.

In this study, the average AHI, as well as the prevalence of the different sub-classes of OSA severity of the two sub-groups of patients, did not present a statistical difference (*p* > 0.05 in each case). Moreover, no differences between mean BMI values in the two groups of patients emerged (*p* = 0.06). This would suggest that the AHI and BMI values found in the two patient sub-groups could not to be considered as potential bias factors of the study. 

What does it mean and how does it help in clinical practice to know the prevalence of POSA in elderly patients?

The gold standard of OSA treatment in elderly patients remains as CPAP ventilatory therapy. This results in a reduction in the AHI and an improvement in nighttime saturation. However, an emerging problem related to ventilatory therapy is that between 30% and 50% of patients do not tolerate the device [40,41,42]. This percentage is today also increasing in patients over 65 years old who tend to accept this device less frequently. For this reason, in the population over 65 years old, new therapies are always considered. Surgical treatment is often not recommended in patients over 65 years old. The effects and complications of sleep surgery in elderly patients has been recently analyzed by Gouveia et al. [43]. Elderly patients had higher rates of wound complications and urinary tract infections as compared with adult patients [43]. On multivariate analysis, age 65 was significantly associated with complications from sleep surgery (odds ratio, 2.35; 95% CI, 1.04–5.35). The possibility of using an oral device (mandibular advancement device) in elderly patients is often limited by the precarious dental conditions of such patients.

Therefore, in elderly patients who refuse CPAP therapy, the possibility of using positional therapy should be considered, particularly in patients with a mild/moderate disease.

This statement is confirmed by the 22.6% of elderly patients classified as APOC 1 (patients that theoretically can be cured with the use of PT alone) and 38.9% of APOC II (patients who can decrease the OSA severity category through treatment with PT) of this study. However, the efficacy and applicability of positional therapy in elderly patients must be confirmed by further research on the topic. A further study is under way to validate the effect of PT therapy in elderly patients.

A limitation of this study is that the Rapid Eye Movement (REM) and non-REM sleep stages were not considered and analyzed. Some studies have shown that the AHI could be worse during the REM phase in supine sleep in comparison to supine non-REM sleep or non-supine REM sleep. However, the largest published studies have found a positional effect during both non-REM and REM sleep, and when authors carefully looked for an interaction between sleep stage and body position, it was not found [44,45,46].

## 5. Conclusions

In this study, there was a high prevalence of elderly patients with POSA, ranging from 20.5% to 49.3% depending on the classification system chosen. No difference between adult and elderly patients with regard to the prevalence of POSA was observed. A significant number of elderly patients could be considered treatable with positional therapy according to the APOC classification.

## Figures and Tables

**Table 1:** Patient’s characteristics and polysomnographic data.

	<65	>65	
**TOTAL NUMBER OF PATIENTS**	**288**	**146**	
**M**	207 (71%)	100 (68.4%)	**chi square** = 0.38 degree of freedom = 1 *p* = 0.5
**F**	81 (28.1%)	46 (31.5%)	
**MIDDLE AGE**	51.4	72.41	**Student’s *t*-test** *t* = −25.5 *p* = 0.0001 degrees of freedom = 432
**BMI**	Mean = 31.1 CI: 30.29–31.82 SD = 6.55 Hi = 41.1 Low = 20.0 Median = 29.8	Mean = 29.9 CI: 28.86–30.89 SD = 4.49 Hi = 42.6 Low = 20.4 Median = 29.2	**Student’s *t*-test** *p* = 0.06 degrees of freedom = 432
**AHI**	Mean = 27.4 CI: 24.92–29.95 SD = 21.6 Hi = 103 Low = 5.00 Median = 20.1	Mean = 28.3 CI: 24.80–31.86 SD = 22.0 Hi = 109 Low = 5.10 Median = 20.9	**Student’s *t*-test** *t* = -0.4 *p* = 0.6 degrees of freedom = 432
**AHI supine position**	Mean = 38.0 CI: 35.04–40.89 SD = 25.3 Hi = 110 Low = 0.00 Median = 30.4	Mean = 37.7 CI: 33.54–41.76 SD = 25.2 Hi = 118 Low = 2.00 Median = 33.1	**Student’s *t*-test** *t* = 0.1 *p* = 0.9 degrees of freedom = 432
**AHI non-supine position**	Mean = 19.9 CI: 17.31–22.41 SD = 21.9 Hi = 93.0 Low = 0.00 Median = 11.4	Mean = 21.7 CI: 18.16–25.33 SD = 22.4 Hi = 116. Low = 0.00 Median = 12.8	**Student’s *t*-test** *t* = -0.8 *p* = 0.4 degrees of freedom = 432
**Percentage of time in supine position**	Mean = 44.8 CI: 41.99–47.70 SD = 23.2 Hi = 100. Low = 5.70 Median = 42.5	Mean = 44.8 CI: 41.99–47.70 SD = 23.2 Hi = 100 Low = 5.70 Median = 42.5	**Student’s *t*-test** *t* = −0.1 *p* = 0.9 degrees of freedom = 432
**Percentage of time in non-supine position**	Mean = 55.1 CI: 52.29–58.00 SD = 23.2 Hi = 94.3 Low = 0.00 Median = 57.5	Mean = 54.9 CI: 50.88–58.90 SD = 27.3 Hi = 97.2 Low = 0.900 Median = 60.8	**Student’s *t*-test** *t* = 0.7 *p* = 0.9 degrees of freedom = 432
**Mean SpO_2_**	Mean = 91.6 CI: 90.92–92.19 SD = 6.29 Hi = 97.0 Low = 67.5 Median = 92.4	Mean = 91.2 CI: 90.33–92.11 SD = 3.34 Hi = 97.0 Low = 70.5 Median = 91.8	**Student’s *t*-test** *t* = 0.6 *p* = 0.5 degrees of freedom = 432
**Total time of sleep(hours)**	Mean = 7.03 SD = 0.837 Hi = 8.80 Low = 6.00 Median = 7.00 Average Absolute Deviation from Median = 0.677	Mean = 7.10 SD = 0.789 Hi = 9.00 Low = 6.00 Median = 7.00 Average Absolute Deviation from Median = 0.580	**Student’s *t*-test** *t* = -0.1 *p* = 0.4 degrees of freedom = 432
**ODI**	Mean = 30.1 CI: 26.41–33.71 SD = 21.9 Hi = 109 Low = 4.40 Median = 22.9	Mean = 30.1 CI: 26.41–33.71 SD = 21.9 Hi = 109 Low = 4.40 Median = 22.9	**Student’s *t*-test** *t* = -0.5 *p* = 0.5 degrees of freedom = 432

Body mass index (BMI), apnea–hypopnea index (AHI), oxygen desaturation index (ODI), confidence interval (CI), standard deviation (SD), high value (Hi), lower value (Low).

**Table 2:** Distribution of patients according to Obstructive Sleep Apnea (OSA) severity sub-classes.

	<65 288 Patients	>65 146 Patients	*p*-Value Chi Square Test
**Mild OSA**	106 (36.8%)	47 (32.1%)	*p* = 0.3 degrees of freedom = 1
**Moderate OSA**	88 (30.5%)	52 (35.6%)	*p* = 0.4 degrees of freedom = 1
**Severe OSA**	94 (32.6%)	47 (32.1%)	*p* = 1 degrees of freedom = 1

**Table 3:** Prevalence of position-dependent OSA (POSA) according to Cartwright, Bignold, and Amsterdam Positional OSA Classification (APOC) classifications.

POSA CLASSIFICATION	<65 Years Old 288 Patients		>65 Years Old 146 Patients		Chi Square Test with Yates Correction *p*-Value Degrees of Freedom
	POSA +	POSA -	POSA +	POSA -	
**CARTRIGHT**	148 (51.3%)	140 (48.6%)	72 (49.3%)	74 (50.7%)	Chi squared = 0.09 degrees of freedom = 1 *p* = 0.7
**BIGNOLD**	66 (22.9%)	222 (77.1%)	30 (20.5%)	116 (79.4%)	Chi squared = 0.2 degrees of freedom = 1 *p* = 0.6
**APOC1**	86 (29.8%)		33 (22.6%)		Chi squared = 2.2 degrees of freedom = 1 *p* = 0.1
**APOC2**	59 (20.4%)		27 (38.9%)		Chi squared = 0.1 degrees of freedom = 1 *p* = 0.7
**APOC3**	20 (6.9%)		8 (5.4%)		Chi squared = 0.14 degrees of freedom = 1 *p* = 0.6
**NON APOC**	123 (42.7%)		78 (53.4%)		Chi squared = 4.05 degrees of freedom = 1 *p* = 0.06
